# HIF-2α/LPCAT1 orchestrates the reprogramming of lipid metabolism in ccRCC by modulating the FBXW7-mediated ubiquitination of ACLY

**DOI:** 10.7150/ijbs.103032

**Published:** 2025-01-01

**Authors:** Mintian Fei, Yi Zhang, Haolin Li, Qili Xu, Yu Gao, Cheng Yang, Weiyi Li, Chaozhao Liang, Baojun Wang, Haibing Xiao

**Affiliations:** 1 Department of Urology, the First Affiliated Hospital of Anhui Medical University, Anhui Medical University, Hefei, Anhui, PR China.; 2 Institute of Urology, Anhui Medical University, Hefei, Anhui, PR China.; 3 Anhui Province Key Laboratory of Urological and Andrological Diseases Research and Medical Transformation, Anhui Medical University, Hefei, Anhui, PR China.; 4 Department of Urology, The 1st Affiliated Hospital of Kunming Medical University, Kunming, 650032, PR China.

**Keywords:** ccRCC, HIF-2α, LPCAT1, FBXW7, Lipid Metabolism and Ubiquitination

## Abstract

The current research revealed a strong link between lipid reprogramming and dysregulated lipid metabolism to the genesis and development of clear cell renal cell carcinoma (ccRCC). Pathologically, ccRCC exhibits a high concentration of lipid droplets within the cytoplasm. HIF-2α expression has previously been demonstrated to be elevated in ccRCC caused by mutations in the von Hippel-Lindau (VHL) gene, which plays a vital role in the development of renal cell carcinoma. Nevertheless, the mechanisms by which HIF-2α influences lipid metabolism reprogramming are unknown. Our investigation demonstrated that HIF-2α directly binds to the promoter region of LPCAT1, promoting its transcription. RNA-seq and lipidomics mass spectrometry studies showed that knocking down LPCAT1 significantly reduced triglyceride production. Research suggests that KD-LPCAT1 involves activation of the NF-κB signaling pathway, which activates F-Box/WD Repeat-Containing Protein 7 (FBXW7). FBXW7, an E3 ubiquitin ligase involved in lipid metabolism, interacts with ATP Citrate Lyase (ACLY) to promote its degradation, lowering fatty acid production and contributing to the lipid content reduction.

## Introduction

RCC is a malignant tumor originating from the renal tubular epithelium 1. The Global Cancer Statistics 2020 report indicates that RCC is the sixth most common malignancy in men and the ninth in women, representing 5% and 3% of cases, respectively 2. RCC comprises approximately 80% to 90% of malignant kidney tumors 3. The most prevalent type of renal cell carcinoma is clear cell renal cell carcinoma (ccRCC), which accounts for about 80% of cases. The name "clear cell" refers to the substantial accumulation of lipids and glycogen in ccRCC cytoplasm, which distinguishes it from other subtypes 4. The aggregation of lipids and glycogen provides adequate energy support during the development of ccRCC. Despite significant advances in understanding the genetic and expression profiles that drive ccRCC, the molecular mechanisms underlying the clear cell phenotype and its relevance remain to be investigated 5.

The mutation of the von Hippel-Lindau (VHL) gene is a hallmark of clear cell renal cell carcinoma (ccRCC) 6. Hypoxia-inducible factors are recognized and bound by VHL, an E3 ubiquitin ligase 7. Mutations or functional loss of VHL elicit aberrant accumulation of downstream hypoxia-inducible factors (HIFs) 8. HIF is the primary transcription factor that coordinates the hypoxic adaptive response, playing a role in processes such as angiogenesis, tumor cell proliferation, cell survival and progression, metastasis, and the transcription of glucose metabolism-related genes during tumor development 9-11. A heterodimeric protein, HIF is composed of a constitutively expressed β and an oxygen-sensitive α component. Three subunits have been identified, namely HIF-1α, HIF-2α, and HIF-3α 12. Among this HIF-2α is overexpressed in ccRCC, contributing to its genesis, progression, and lipid accumulation 13.

A notable characteristic of ccRCC is the accumulation of lipid droplets within cells, along with elevated contents of fatty acids, cholesterol and triglycerides in ccRCC tissues compared to adjacent non-cancerous tissues 14. Previous studies indicate that HIF-2α transcriptionally regulates Perilipin 2 (PLIN2), thereby stabilizing endoplasmic reticulum homeostasis and promoting tumor growth 13. HIF-2α also inhibits carnitine palmitoyltransferase 1A (CPT1A), which results in a decrease in mitochondrial transport of fatty acids and in an increase in lipid droplet incorporation 5. Although aberrant lipid accumulation and HIF-2α activation are well-recognized features of dysregulation in ccRCC, the underlying mechanisms require further investigation.

LPCAT1 is a crucial enzyme in phospholipid synthesis and remodeling, regulating the fatty acyl composition of phospholipids by incorporating fatty acyl chains into the sn-2 position of phosphatidylcholine, thus completing phospholipid remodeling 15. Li *et al.* demonstrated that LPCAT1 promotes membrane phospholipid saturation and lower polyunsaturated fatty acid levels through the Lands cycle. This reduces membrane damage and ferroptosis caused by phospholipid peroxidation, facilitating tumor growth 16. Furthermore, LPCAT1 can regulate the cell cycle via phospholipid metabolism; knocking down LPCAT1 causes G0-G1 phase cell arrest in ccRCC cells 17. In addition to its role in phospholipid metabolism, LPCAT1 can reprogram cholesterol metabolism by regulating the cholesterol synthesis enzyme SQLE, enhancing the evolution of esophageal squamous cell carcinoma 18. Recently, it was discovered that LPCAT1 can influence de novo lipogenesis-related metabolic pathways in hepatocellular cancer 19. Therefore, it is important to investigate whether LPCAT1 regulates fatty acid metabolism in ccRCC, as well as the mechanisms that influence tumor formation.

## Materials and Methods

### Human samples

Tissue samples, including ccRCC and adjacent non-tumor tissues, were obtained from the First Affiliated Hospital of Anhui Medical University. The collected samples were divided into three groups upon collection: one for paraffin embedding for immunohistochemistry, another for protein extraction for Western blot analysis, and the last for RNA extraction, with samples immediately immersed in RNA Later solution. The latter two groups of samples were stored in a -80°C freezer. The type of tumor for each tissue sample was confirmed by pathological diagnosis, and the use of the tissue samples was consented to by the patients. This study adhered to the Declaration of Helsinki standards and received approval from the Ethics Committee of Human Research at The First Affiliated Hospital of Anhui Medical University (PJ 2023-08-38).

### Cell culture and reagents

All cells were sourced from Procell (Wuhan, China). The culture conditions were maintained at 37°C with 5% CO2. The specific culture media used for each cell line were as follows:786-O and OS-RC-2: RPMI 1640 medium with 10% FBS.A-498 and ACHN: MEM medium containing 10% FBS, Caki: McCoy's 5A medium containing 10% FBS. In addition to these renal carcinoma cell lines, the following were also used: HK-2: MEM medium containing 10% FBS. Human embryonic kidney cells (293-T): High-glucose DMEM medium containing 10% FBS.

### Lentivirus, plasmids and siRNA

The lentiviruses used for knocking down HIF-2α and LPCAT1 were obtained from GeneChem, while the lentivirus for overexpressing ACLY was prepared by TSINGKE Biological Technology. Flag-FBXW7 and MYC-ACLY was purchased from Youbio. All transfection procedures were carried out according to the protocols provided by the respective companies. The sequences for each gene are provided in the supplementary table. Transient transfection was carried out using Lipofectamine 2000 (Invitrogen; Thermo Fisher Scientific, Inc.) according to the protocols.

### RNA isolation and qRT-PCR

Total RNA was extracted using TRIzol reagent (Invitrogen, Carlsbad, CA), according to the manufacturer's protocol provided by the manufacturer. Subsequently, qRT-PCR was performed using the ABI7500 platform (Thermo Fisher Scientific, Massachusetts, USA), according to the manufacturer's instructions. Total RNA was converted to cDNA using the PrimeScriptTM RT Reagent Kit (Takara, Kusatsu, Japan). Quantitative PCR was performed with SYBR Green Master Mix (Takara, Kusatsu, Japan). Gene expression levels were normalized to GAPDH and ACTIN expression. Sangon Biotech offer the primers (Shanghai, China). Primer sequences are available in the supplementary tables.

### Western blotting assay

Proteins were extracted using RIPA lysis buffer (Beyotime Biotech, Jiangsu, China) supplemented with phosphatase and protease inhibitors. Protein concentrations were quantified using spectrometer (A280). Approximately 20 μg of protein was separated by electrophoresis and transferred onto a methylcellulose membrane. The membranes were blocked with non-fat dry milk at room temperature for one hour, followed by overnight incubation at 4°C with specific primary antibodies: HIF-2α (Novus, #NB100-122, 1:1000); LPCAT1 (Proteintech, #16112-1-AP, 1:1000); FBXW7 (Proteintech, #28424-1-AP, 1:500); NF-κB p65 (CST, #8242, 1:1000); Phospho-NF-κB p-p65 (CST, #3033, 1:1000); ACLY (Abcam, ab40793, 1:10000); FLAG-tag (Merck, #F1804, 1:1000); MYC-tag (Proteintech, #16286-1-AP, 1:1000). Following primary antibody incubation, the membranes were treated with secondary antibodies at room temperature for 1 hour. The membranes were washed with Tris Buffered Saline with Tween 20 (TBST) and developed using enhanced chemiluminescence detection.

### IHC assay

Tissue microarrays were constructed from ccRCC samples that had been fixed in 4% formalin and embedded in paraffin. After sectioning, the tissues were subjected to antigen retrieval, blocked with 1% BSA in PBST buffer at room temperature for 2 hours, and subsequently incubated overnight at 4°C with specific primary antibodies. Following three washes, the sections were treated with HRP-conjugated polymer secondary antibodies at room temperature. The slides were then stained with 3,3'-diaminobenzidine (DAB) substrate and counterstained with hematoxylin. Finally, a pathological imaging system was employed to scan the prepared slides.

### Cell proliferation, cell migration, invasion and wound healing

Cell Proliferation: 786-O and A-498 were resuspended in medium containing 5% FBS at a density of 3000 cells per well. The Cell Counting Kit-8 was used to measure cell proliferation (GlpBio, #GK10001) on days 0, 1, 2, 3, and 4. Concentration for Cell Counting Kit-8: serum-free medium = 1:10 and then Incubating in a 5% CO2 incubator for 1 hour). For migration and invasion assays, 786-O and A-498 cells were resuspended in serum-free medium at densities of 5000 cells/mL for migration and 3000 cells/mL for invasion. Cells were seeded in the upper chamber of a transwell insert with 8 μm pores, while the lower chamber was filled with medium containing 10% FBS. The cells were incubated for 36 hours at 37°C with 5% CO2. After incubation, crystal violet staining was done on the cells after they had been fixed with 4% paraformaldehyde. Quantification of stained cells was then performed under a microscope. Cells were seeded in a 6-well plate and pretreated with serum-free medium upon reaching 80% confluence. Upon reaching 80% confluence, a sterile 200 μL pipette tip was employed to scratch the cell monolayer, creating a wound. Wound healing was observed and recorded at 0 hours and 24 hours using a microscope 20.

### Oil red O staining

Cells were seeded in a 6-well plate and stained with Oil Red O using the Oil Red O Stain Kit (Solarbio, G1262) upon reaching 50% confluence. Washing the cells twice with PBS and fix them with fixing buffer for 30 minutes. Rinse the cells with distilled water twice, followed by a 5-minute incubation in 60% isopropanol. Incubate with freshly prepared Oil Red O staining solution for 20 minutes. Add Mayer hematoxylin staining solution for 2 minutes. Discard the stain and wash 3 times. Finally, record the results using a microscope.

### Lipid droplet staining and quantification

Lipid droplets were stained using BODIPY 493/503 (Invitrogen, #D2191). Seeding cells onto sterile glass coverslips and culture until 50% confluence. Rinse cells twice with PBS. For 30 minutes, fix cells with 4% paraformaldehyde at room temperature. Stain lipid droplets with 2 μM BODIPY 493/503 for 15 minutes, then wash cells thrice with PBS. Stain nuclei with 10 μg/ml Hoechst 33342 (MCE, HY-15559) for 5 minutes, then wash cells three times with PBS. Record stained cells using a microscope. For flow cytometry analysis of BODIPY 493/503, fixative treatment is not required; otherwise, follow the same staining procedure.

### RNA sequencing

Total RNA was extracted with TRIzol (Ambion). RNA integrity was assessed using an Agilent 2100 Bioanalyzer (Agilent Technologies, Santa Clara, CA, USA). Samples with RNA Integrity Number (RIN) of 7 or higher were selected for further analysis. Library construction followed the manufacturer's instructions using the TruSeq Stranded mRNA LTSample Prep Kit (Illumina, San Diego, CA, USA). The libraries were sequenced using an Illumina platform (HiSeq 2500 or HiSeq X Ten). Whole transcriptome sequencing was conducted by Frasergen (Wuhan, China).

### LC/MS (Liquid Chromatography/Mass Spectrometry)

Cells from two 10 cm dishes of 786-O and 786-sh-LPCAT1 were digested with trypsin, washed twice with PBS, and then rapidly frozen in liquid nitrogen. To extract lipids, 1 mL of internal standard lipid extraction solution was added. Following the vortex of 15 minutes, 200 μL of water were added and vortexed for 1 additional minute. The sample was centrifuged at 12,000 rpm for 10 minutes at 4°C. The upper layer was transferred to a new centrifuge tube and evaporated to dryness. The dried sample was reconstituted with 200 μL of a lipid reconstitution solution (acetonitrile: isopropanol = 1:1, V/V), vortexed for 3 minutes, and centrifuged at 12,000 rpm for 3 minutes. The supernatant was collected and analyzed using an Ultra Performance Liquid Chromatography (UPLC) system (ExionLC AD, https://sciex.com.cn/) coupled with Tandem Mass Spectrometry (MS/MS). This PCA was conducted using the prcomp function in R (www.r-project.org). with unit variance scaling. Hierarchical Cluster Analysis (HCA) and Pearson Correlation Coefficients (PCC) between samples were both visualized as heatmaps, with HCA including dendrograms. PCC calculations utilized the cor function in R. LC/MS was conducted by Frasergen (Wuhan, China).

### ChIP assays

ChIP assays were performed using the 786-O cell line. Each immunoprecipitation sample required approximately two 15 cm dishes of cells. The extraction and immunoprecipitation were carried out following the SimpleChIP® Enzymatic Chromatin IP Kit (Magnetic Beads) protocol (CST, #9003). The lysates were immunoprecipitated with antibodies against HIF-2α (Novus), normal rabbit IgG, and VEGFR antibodies. The DNA samples purified by the kit were analyzed using qRT-PCR to detect the binding of HIF-2α to the LPCAT1 promoter region and to assess its impact on transcription. Product from qRT-PCR amplification were verified using gel electrophoresis.

### Dual luciferase reporter assay

HEK293-T cells were seeded at a density of 1 × 10^5 cells per well in 24-well plates with three replicates per group. When the cells reached 60% confluence, they were co-transfected with HIF-2α plasmid, LPCAT1 promoter reporter plasmids, and various mutant promoter region plasmids. The transfection was performed for 48 hours. After incubation, the Dual-Luciferase® Firefly & Renilla Assay Kit (UElandy) was used to measure luciferase activity. The luciferase activity data were analyzed using bioinformatics tools.

### Co-immunoprecipitation (Co-IP) assay

Transfect HEK293-T cells in a 6-well plate with either FLAG-FBXW7 or MYC-ACLY plasmids for 48 hours until cell confluence reaches 90%-100%. Washing the cells twice with cold PBS, then lyse them in 120 μL of RIPA lysis buffer with protease and phosphatase inhibitors at 4°C for 20 minutes. Collecting the lysate using a cell scraper and centrifuge at 12,000 g and 4°C for 15 minutes. Transfering the supernatant to a fresh EP tube and incubate overnight with 1-2 µg of IgG or antibody against the target protein. Following an overnight rotation of the lysate at 4°C, introduce 20 µL of IPKine™ Protein A/G Magnetic Beads (Abbkine, BMR2080). Washing the beads with IP lysis buffer, then boil in 2× SDS protein loading buffer at 100°C for 5 minutes. Loading 10 µL of the sample onto an SDS-PAGE gel and perform Western blot analysis.

### Mouse tumor growth

To culture the 786-O cells in a 5%, 37°C CO2 incubator. Digest the cells with trypsin, wash with PBS, centrifuge, discard the supernatant, and collect the 786-O cells. For the subcutaneous tumor model, adjust the cell density to 1 × 107 and add 100 μL of Matrigel 26. BALB/c-Nude mice, 5 weeks old, purchased and acclimated for 1 week in an animal laboratory. Disinfect the mice and anesthetize them. Use povidone-iodine to disinfect the skin. Subcutaneously inject 100 µL of cell suspension into the dorsal side of the mice to establish a tumor model. The tumor measurement method is referenced from this article 27. After euthanizing the mice, collect the tumor tissues and measure the triglyceride content. Anhui Medical University's Animal Care and Use Committee approved all animal experimentation protocols (LISC20242183).

### Statistical analyses

All data analyses were performed using GraphPad Prism 9 and R 4.3.2, with a threshold of p < 0.05 set to determine statistical significance. Continuous data were analyzed using Student's two-tailed t-test, while ANOVA was employed for comparisons involving more than two groups. Data are presented as mean ± SD, with statistical significance indicated as ***p < 0.001, **p < 0.01, and *p < 0.05.

## Results

### VHL-HIF-2α axis can regulate lipid metabolism in ccRCC

**Figure 1:**
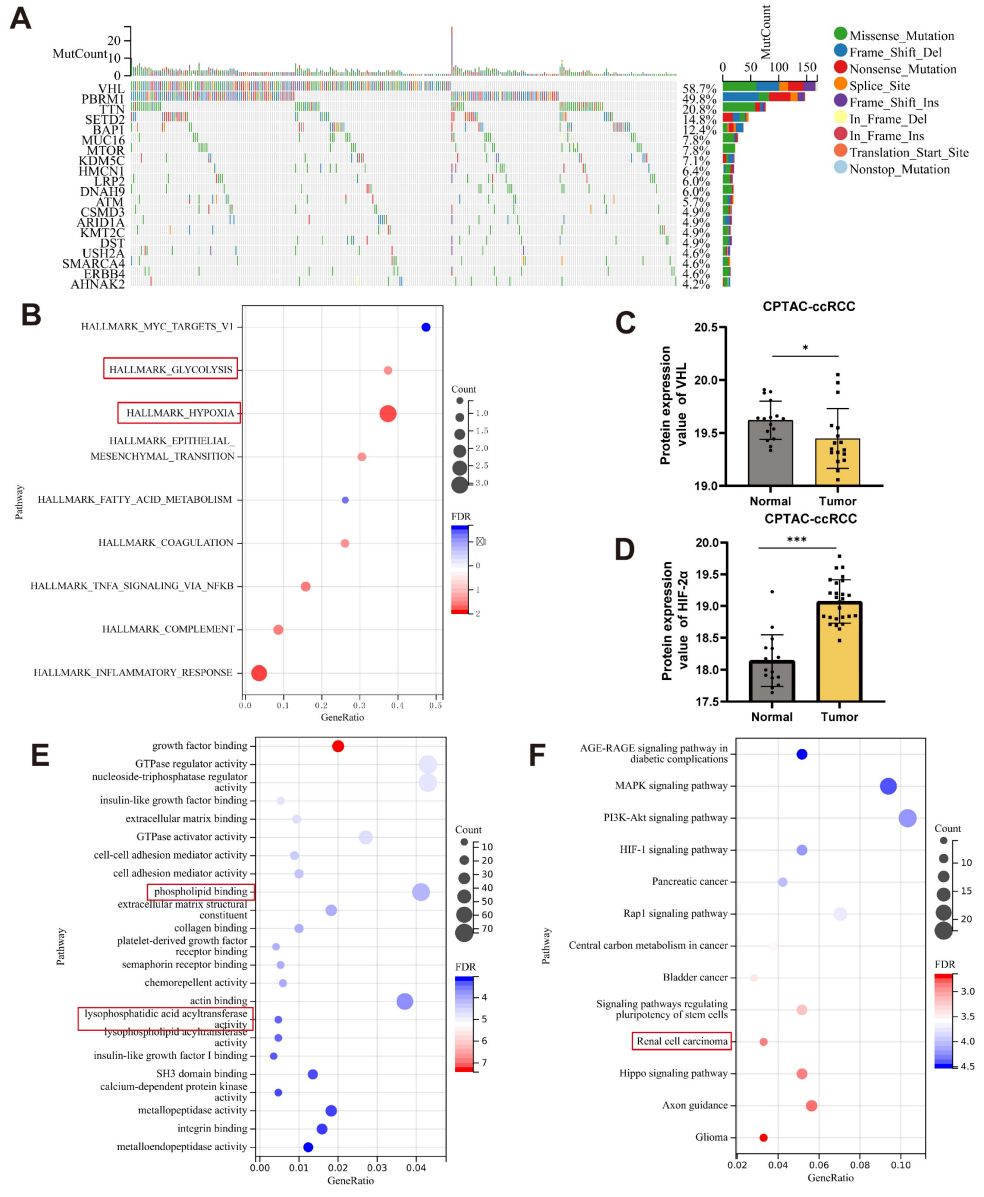
VHL-HIF-2α axis can regulate lipid metabolism in ccRCC. **A**. The waterfall plot of mutated genes in ccRCC analyzed from the TCGA database, generated using the Sangerbox online platform **B**. Pathway enrichment analysis of the VHL mutant gene set. **C, D**. Analysis of VHL and HIF-2α gene expression levels in the ccRCC dataset from the CPTAC database. **E**. GO enrichment analysis using RNA-seq data from KO-HIF-2α.** F**. KEGG pathway enrichment analysis for PT2385.

Previous research has revealed that von Hippel-Lindau (VHL) mutations are typically found in RCC. According to the TCGA-KIRC dataset, VHL mutations are the most common among various mutated genes (Fig. 1A). As an E3 ubiquitin ligase, VHL regulates the expression of other genes through its binding activity. Pathway enrichment analysis of the dataset revealed that VHL is associated with hypoxia and glycolysis (Fig. 1B). VHL regulates several signaling pathways, including the VHL-HIF-2α axis, which is critical for RCC development and progression. The Clinical Proteomic Tumor Analysis Consortium (CPTAC) found that VHL expression levels were lower in ccRCC tumor tissues than in normal tissues (Fig. 1C). In contrast, HIF-2α expression is elevated, suggesting a connection between VHL and HIF-2α in ccRCC. To further explore the regulatory mechanisms of HIF-2α in ccRCC, we analyzed the HIF-2α knockout (KO-HIF-2α) dataset (GSE153711) from the GEO database. The research demonstrated that knocking out HIF-2α may impair lysophosphatidic acid acyltransferase activity and phospholipid binding (Fig. 1E). Furthermore, KEGG enrichment analysis of 786-O cells treated with an HIF-2α antagonist PT2385 (GSE153711) revealed a relationship with renal cell cancer (Fig. 1F). A recent study indicates that HIF-2α can impact lipid accumulation in ccRCC 5. We hypothesize that the VHL-HIF-2α axis regulates lysophosphatidic acid acyltransferase activity, affecting lipid reprogramming in ccRCC.

### HIF-2α transcriptionally regulates LPCAT1

**Figure 2:**
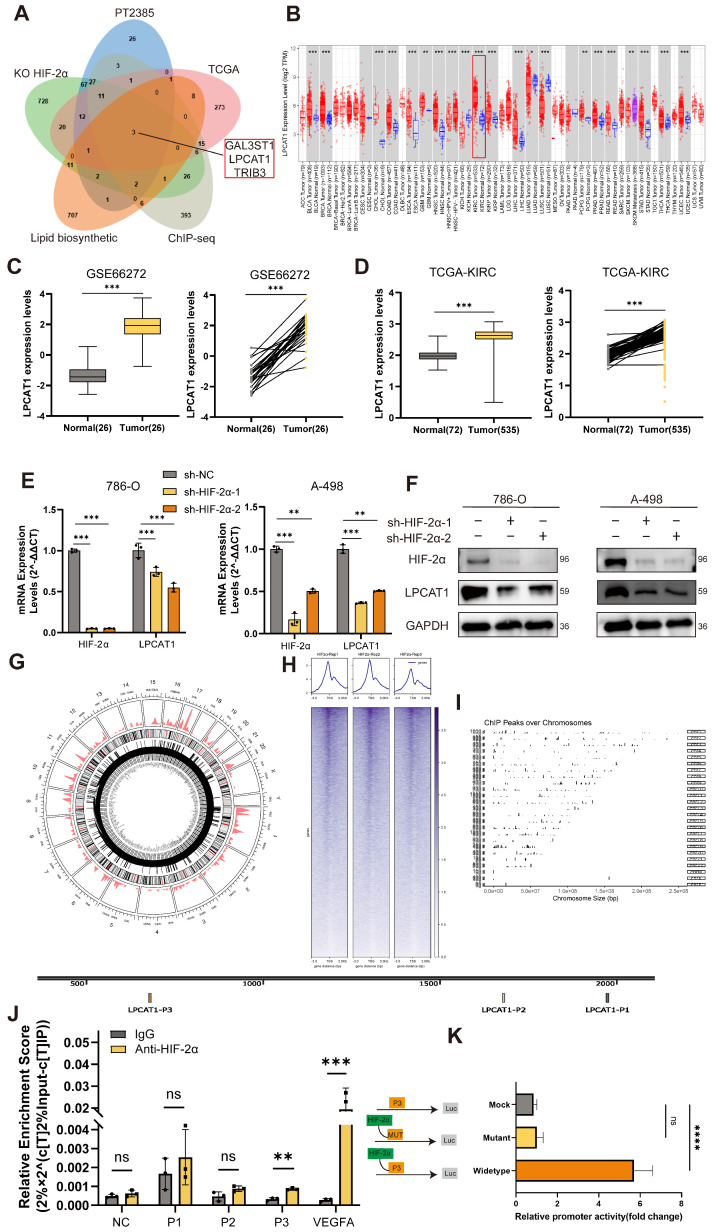
HIF-2a transcriptionally regulates LPCAT1. **A**. Venn diagram was drawn using four independent datasets, including KO-HIF-2α, PT2385, TCGA, ChIP-seq, Lipid biosynthetic to show downregulated differentially expressed genes, including LPCAT1 and TRIB3.** B**. Pan-cancer analysis of the TCGA dataset and differential expression of LPCAT1 in tumor and normal tissues. **C, D**. GEO and TCGA datasets analysis the LPCAT1 expression leves in tumor and normal. **E**. qRT-PCR shows the changes in mRNA levels of HIF-2α and LPCAT1 after knockdown of HIF-2α. **F**. Western blot verifies the effect of HIF-2α knockdown and shows the changes in LPCAT1 protein levels. **G**. The circos plot displays the chromatin regions where HIF-2α may bind and the binding intensity. **H**. Reads distribution around the transcription start site (TSS). **I**. Chromosomes of ChIP peaks by ChIP-seq using the HIF-2α antibody (GSE183900). **J**. Chromatin immunoprecipitation (ChIP) was performed using an antibody against HIF-2α with chromatin extracted from 786-O cells. Prior to immunoprecipitation, 2% of the cell lysate was collected as input then IgG as a negative control. After purification, qRT-PCR and PCR were performed using primers designed based on the predicted three potential HIF-2α binding sites. The experiments were independently repeated three times. **K**. Based on the sequence of the P3 site, an overexpression plasmid for the P3 site and a mutant plasmid for P3, along with an HIF-2α plasmid, were designed. Incubated 293-T cells for 24 hours with the transfected plasmids. Then, the activity of LPCAT1 was detected using a dual-luciferase reporter assay kit. (Mock: transfected with P3 plasmid, Mutant: transfected with HIF-2α plasmid + P3 mutant plasmid, Wildtype: transfected with HIF-2α + P3 plasmid).

To investigate how HIF-2α influences lipid metabolism, we utilized differentially expressed genes (DEGs) from the KO-HIF-2α dataset in the GEO database and the HIF-2α antagonist PT2385 (GSE13711). In addition, we incorporated genes significantly expressed in renal cell carcinoma tissues from TCGA, together with HIF-2α ChIP-seq data (GSE207575) 21. To investigate the relationship between HIF-2α and lipid synthesis, we accessed gene sets from MSigDB. A Venn diagram of these gene sets revealed three common genes: GAL3ST1, LPCAT1, and TRIB3. Previous research has found that TRIB3 is increased in ccRCC and is linked to lipid homeostasis and synthesis 22. Furthermore, the VHL-HIF axis regulates GAL3ST1 in ccRCC, which affects the tumor's immune evasion capabilities. To explore the link between HIF-2α and lipid metabolism, we focused our investigation on LPCAT1(Fig.2A). Pan-cancer investigation using TIMER2.0 online platform revealed that LPCAT1 expression is elevated in various types of tumors, particularly in ccRCC, where expression levels in tumor tissues greatly exceed those in normal tissues (Fig.2B). Our next step was to compare LPCAT1 gene expression levels between tumors and normal tissues using the GEO dataset GSE66272 and the TCGA-KIRC dataset, which showed significantly elevated LPCAT1 expression in tumor tissues (Fig. 2C-D). To study the impact of HIF-2α on LPCAT1, we created a cell line with stable knockdown of HIF-2α. The expression of LPCAT1 at both the mRNA and protein levels was significantly decreased when HIF-2α was knocked down (Fig. 2E-F). The circos plot shows HIF-2α binding peak across multiple chromosomes (Fig. 2G). We investigated the HIF-2α ChIP-seq dataset (GSE207575) and anticipated that HIF-2α may bind to the promoter region of LPCAT1, adapting its activity. The peaks on the chromosomes showed different peak values (Fig. 2H). The horizontal axis represents chromosome size, the right vertical axis indicates chromosome number, and the left vertical axis shows peak values for each chromosome (Fig. 2I). To investigate how HIF-2α regulates LPCAT1, we evaluated putative hypoxia response elements (HREs) in the promoter region and discovered three binding sites. To determine whether HIF-2α regulates LPCAT1 transcription on 786-O chromatin, we performed a ChIP assay, which demonstrated that HIF-2α enhances LPCAT1 transcription by directly binding to its P3 region (Fig. 2J). Additionally, we employed qRT-PCR to amplify the DNA, followed by gel electrophoresis, which confirmed the qRT-PCR results (Supplementary Figures S1A). To provide more evidence of the binding between HIF-2α and LPCAT1, we used a dual luciferase reporter assay with mutant plasmids targeting the P3 location. We transfected 293-T cells with wild-type or mutant LPCAT1 and HIF-2α overexpression plasmids. Mutations in the HREs sites dramatically reduced HIF-2α-induced LPCAT1 promoter activity (Fig. 2K). These findings indicate that HIF-2α regulates LPCAT1 expression in ccRCC by binding to its promoter.

### LPCAT1 promoted the progression of ccRCC

**Figure 3:**
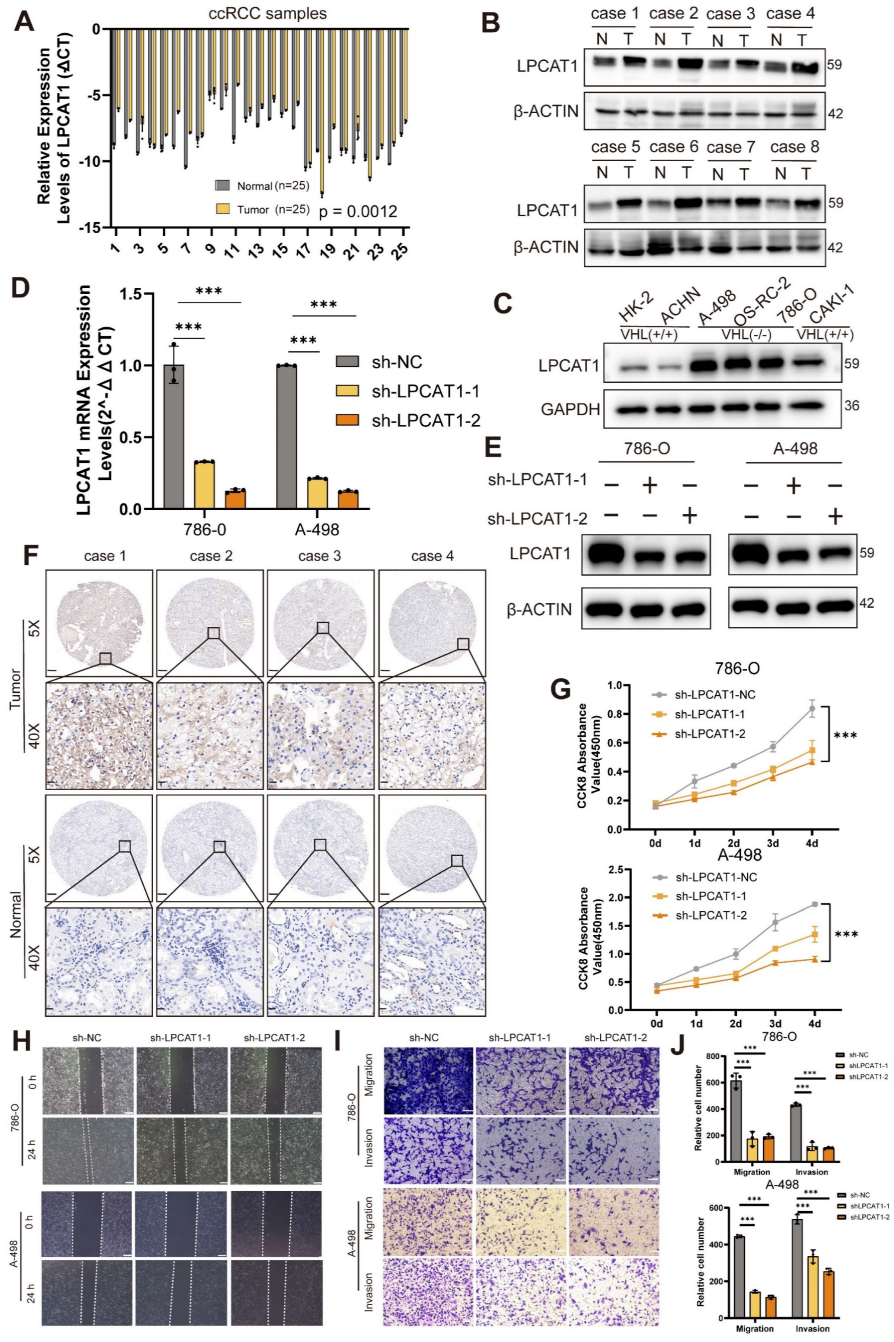
LPCAT1 promoted the progression of ccRCC. **A**. qRT-PCR analysis of LPCAT1 mRNA levels in tumor tissues compared to adjacent normal tissues. **B**. Western blot assay of LPCAT1 protein levels in renal cell carcinoma compared to adjacent normal tissues. **C**. Western blot assay of LPCAT1 expression in RCC cell lines, with HK-2 as a normal cell reference. **D**. qRT-PCR shows the changes in mRNA expression levels in A-498 and 786-O cells after knockdown of LPCAT1 using sh-RNA. **E**. Western blot assay the changes in LPCAT1 protein levels in A-498 and 786-O cells after knockdown of LPCAT1 using sh-RNA. **F**. Immunohistochemical analysis of LPCAT1 in ccRCC samples and corresponding adjacent normal tissues. (Scale: 200μm, magnified view: 20μm). **G**. CCK-8 assay shows that the proliferation rate of ccRCC cells significantly slows down after knockdown of LPCAT1 (n=3). **H**. The wound healing assay shows that the migration ability of A-498 and 786-O cells is reduced 24 hours after knockdown of LPCAT1. **I**. Transwell assay shows that the migration and invasion abilities of renal cancer cells are weakened after knockdown of LPCAT1 (scale=100μm). **J**. Counting cells in transwells.

We conducted qRT-PCR and Western blot assays on ccRCC samples to compare LPCAT1 expression between tumor and normal tissues. LPCAT1 mRNA and protein levels were significantly elevated in tumor than normal tissues (Fig. 3A-B). The protein quantification analysis results showed the same trend (Supplementary Figures S1B). The IHC assay also revealed a significant increase in LPCAT1 expression in tumor tissues (Fig. 3F). The positive rate of LPCAT1 in tumor tissues is significantly higher than in normal tissues (Supplementary Figures S1C). To determine the role of LPCAT1 in RCC, we performed *in vitro* studies. We utilized a renal cancer cell line model, comparing LPCAT1 expression in three VHL-mutant cell lines (A-498, 786-O, and OS-RC-2) against the HK-2 control. LPCAT1 was significantly expressed in all three VHL-mutant cell lines (Fig. 3C). As a result, we chose 786-O and A-498 as cell models for further investigation. First, we used short hairpin RNA (shRNA) to knock down LPCAT1 in the 786-O and A-498, and we validated the knockdown effectiveness with qRT-PCR and western blot assays (Fig.3D -E). LPCAT1 knockdown significantly reduced the growth of both 786-O and A-498 cells (Fig. 3G). The wound healing and transwell assays revealed that LPCAT1 enhances invasion and migration capacities (Fig. 3H-I). The number of migrating and invading cells reduces as LPCAT1 is knocked down (Fig. 3J). In general, LPCAT1 promotes proliferative, migratory, and invasion activities of ccRCC.

### LPCAT1 deficiency alleviates lipid accumulation in ccRCC

**Figure 4:**
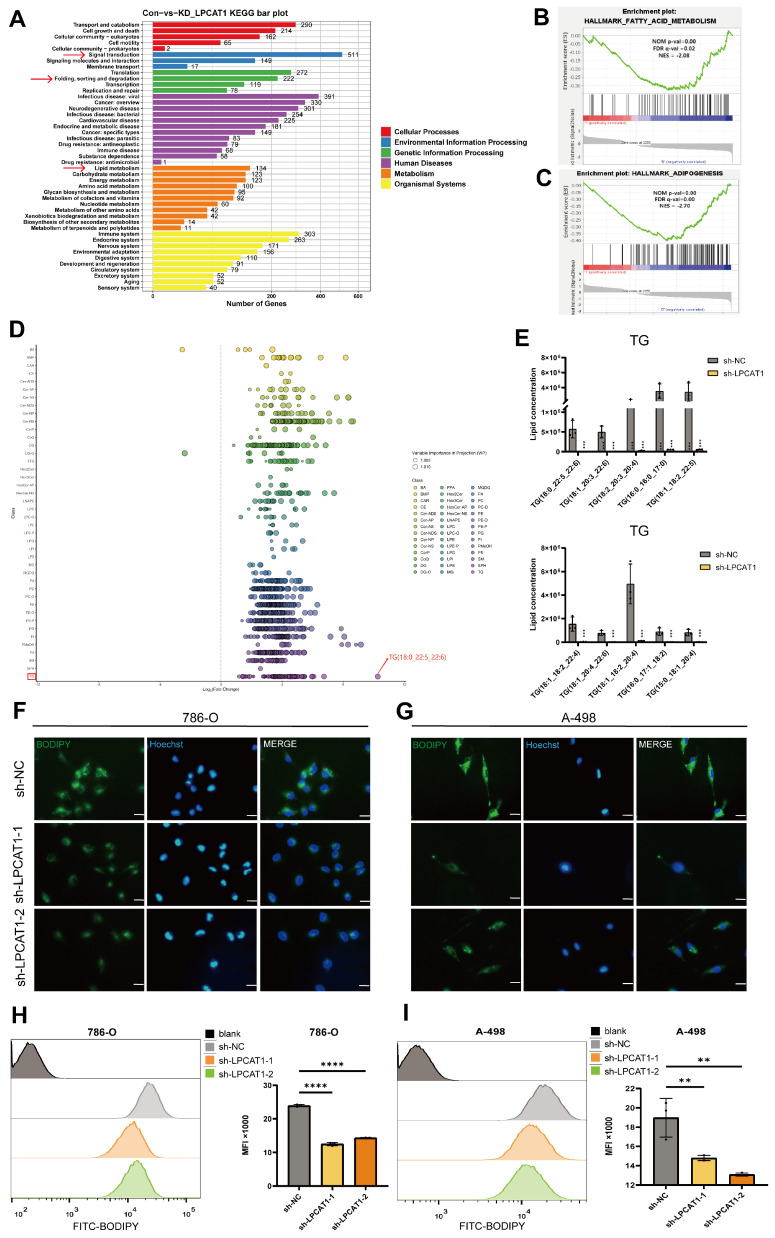
LPCAT1 promotes lipid accumulation in ccRCC by regulating triglyceride metabolism. **A**. Transcriptomic analysis of differentially expressed genes identified by KEGG enrichment analysis after knockdown of LPCAT1 results, with sh-NC as the control. **B, C**. GSEA enrichment results based on transcriptomic differentially expressed genes. **D**. Lipidomic analysis of sh-LPCAT1 compared to sh-NC showing changes in fatty acids (FA), glycerolipids (GL), glycerophospholipids (GP), sphingolipids (SP), and sterols (ST) isoprenoids (PR). **E**. The ten most significantly altered TG components in the LC/MS results. **F, G**. Cells were treated with 2μM bodipy 493/503 for 15 minutes, followed by 10μg/ml Hoechst 33342 to stain the nuclei for 5 minutes, and photographed under a fluorescence microscope (scale=20μm). **H, I**. Cells were treated with 2μM BODIPY493/503 for 15 minutes, then digested with trypsin and analyzed by flow cytometry.

Investigating the potential role of LPCAT1 in ccRCC, we conducted RNA sequencing. KEGG enrichment analysis revealed a significant relationship between LPCAT1 and lipid metabolism, signal transduction, and protein degradation (Fig. 4A). Notably, the top ten pathways identified through GSEA data indicated the strongest correlations with fatty acid metabolism and adipogenesis, suggesting that LPCAT1 may alter lipid droplet accumulation in ccRCC via regulating fatty acid metabolism (Fig. 4B-C) Subsequently, we used a fatty acid assay kit to assess sh-LPCAT 786-O and A-498 cells. It was found that after knocking down LPCAT1, the intracellular fatty acid content significantly decreased (Supplementary Figures S1D). To explore the impact of LPCAT1 on lipid metabolism and the underlying molecular mechanisms in ccRCC, we performed lipidomics mass spectrometry analysis following LPCAT1 knockdown. We examined fatty acids (FAs), glycerophospholipids (GPs), glycerolipids (GLs), sphingolipids (SPs), and sterols (STs) in 786-O cells with and without LPCAT1 knockdown. Statistical analysis of the top five components with significant changes following sh-LPCAT1 treatment (Supplementary Figures S1E). Heatmap showed a significant decrease in various lipid components upon LPCAT1 knockdown (Supplementary Figures S1F). The results revealed a significant decrease in TG content. (Fig. 4E), with the most notable alteration in TG (18:0_22:5_22:6) (Fig. 4D). We performed Oil Red O (Supplementary Figures S1G) and BODIPY 493/503 staining to detect alterations in neutral lipids in sh-NC, sh-LPCAT1-1, and sh-LPCAT1-2. After knocking down LPCAT1, both lipid droplets and neutral lipid concentrations decreased significantly (Fig. 4F-G). Flow cytometry analysis of BODIPY 493/503-treated cells revealed substantial differences in the knockdown group. Collectively, these findings suggest that LPCAT1 may promote lipid accumulation in ccRCC (Fig. 4H-I). Existing research indicates that LPCAT1 is strongly related to phospholipid metabolism and is an important gene in regulating phospholipid production and composition 23. However, its involvement in controlling triglyceride metabolism has been not much investigated. Therefore, we aim to further investigating the mechanism of LPCAT1 on the metabolism of triglycerides in ccRCC.

### LPCAT1 regulates the expression of FBXW7 by NF-κB signaling pathway

**Figure 5:**
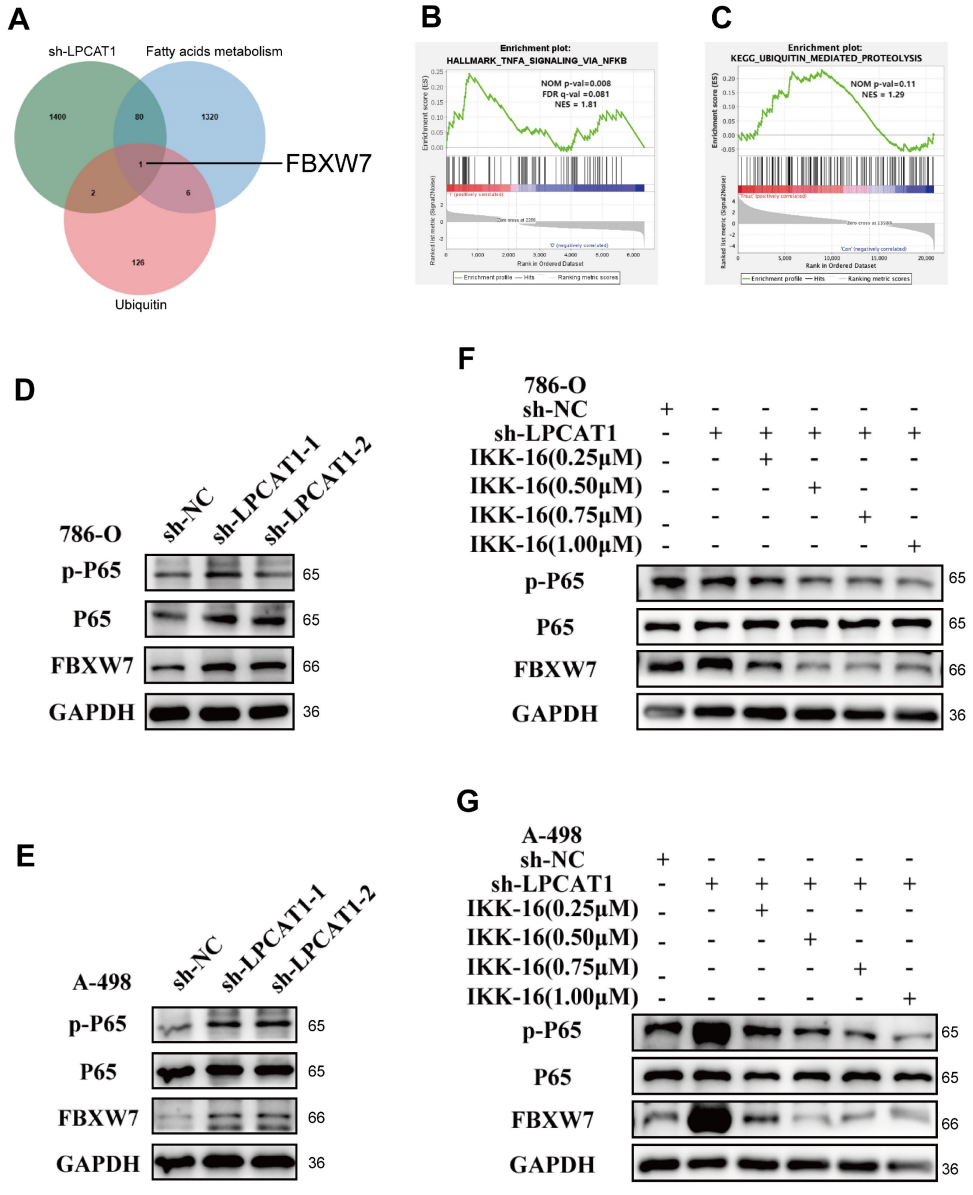
LPCAT1 can influence the expression of FBXW7 by regulating the NF-κB signaling pathway. **A**. Venn diagram of upregulated genes from the differentially expressed genes in sh-LPCAT1 and the gene sets of fatty acids and ubiquitin from GSEA. **B, C**. Analysis of differentially expressed genes from transcriptomic sequencing of sh-LPCAT1 shows that the NF-κB signaling pathway and the process of ubiquitination are enhanced after LPCAT1 knockdown. **D, E**. Verification of the expression of the NF-κB signaling pathway and FBXW7 in786-O and A-498, after LPCAT1 knockdown. **F, G**. Treatment of 786-O and A-498 cells with 0.25μM, 0.50μM, 0.75μM, and 1.00μM of the NF-κB signaling pathway inhibitor IKK-16 to verify the changes in FBXW7 protein expression when the NF-κB signaling pathway is inhibited.

RNA sequencing analysis of sh-LPCAT1 and sh-NC cells uncovered knocking down LPCAT1 increased ubiquitination and activated the NF-κB signaling pathway in 786-O (Fig. 5B-C). Previous research has demonstrated that LPCAT1 enhances carnitine palmitoyltransferase 1 (CPT1) degradation, thereby regulating phospholipid remodeling 24. Consequently, we analyzed ubiquitination and fatty acid metabolism-related gene sets in MSigDB, intersecteing them with differentially expressed genes in sh-LPCAT1 cells, which identified a single relevant gene: FBXW7 (Fig. 5A). LPCAT1 knockdown in 786-O and A-498 cell lines resulted in increased NF-κB signaling and elevated FBXW7 expression (Fig. 5D-E). Additionally, KD-LPCAT1 in 786-O and A-498 cell lines with IKK-16, an NF-κB pathway inhibitor, at dosages of 0.25 μM, 0.5 μM, 0.75 μM, and 1 μM. Inhibition of the NF-κB signaling pathway drastically lowered FBXW7 expression, as demonstrated by Western blot experiments (Fig. 5F-G). These findings indicate that LPCAT1 regulates FBXW7 expression through the NF-κB signaling pathway.

### Overexpression of FBXW7 reduces the expression of ACLY

**Figure 6:**
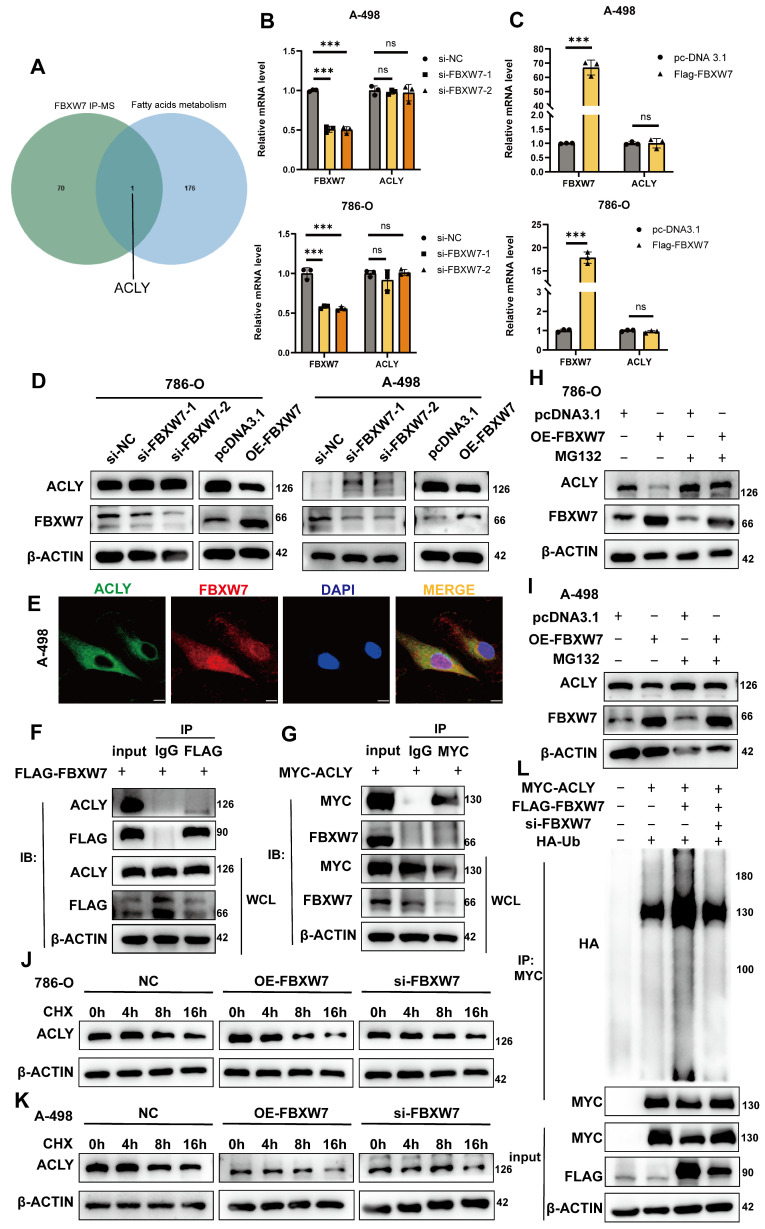
FBXW7 can promote the degradation of ACLY. **A**. Venn diagram analysis of differential proteins from FBXW7 IP-MS and the gene set of fatty acid metabolism pathways. **B**. qRT-PCR detection of changes in mRNA levels of FBXW7 and ACLY in 786-O and A-498 cells after FBXW7 knockdown using si-RNA. **C**. qRT-PCR detection of changes in mRNA levels of FBXW7 and ACLY in 786-O and A-498 cells after FBXW7 overexpression. **D**. Western blot assay detection of changes in ACLY expression in 786-O and A-498 cells after FBXW7 knockdown/overexpression. **E**. Confocal microscopy observation of A-498 cells stained with FLAG and MYC antibodies. Green: MYC-ACLY, Red: FLAG-FBXW7, Blue: DAPI. (scale=10μm). **F**. FLAG-FBXW7 was transfected into 293-T cells for 48 hours, and cell lysates were collected after 48 hours. Using IgG and FLAG antibodies, immunoprecipitation and Western blotting were conducted. **G**. MYC-ACLY were transfected into 293-T cells for 48 hours, and cell lysates were collected. Immunoprecipitation was performed with IgG and MYC antibodies followed by Western blot analysis. **H, I**. Western blot assay of 786-O and A-498 cells with/without MG-132 treatment for 24 hours after different transfection treatments. **J, K**. 786-O and A-498 cells were transfected with FLAG-FBXW7/si-FBXW7 and treated with Cycloheximide for 0h, 4h, 8h, and 16h, followed by collection of cell lysates and Western blot analysis to observe changes in ACLY expression. **L**. 293-T cells were transfected with/without MYC-ACLY, FLAG-FBXW7, si-FBXW7, HA-Ub for 48 hours, and cell lysates were collected. Immunoprecipitation was performed with MYC antibodies to observe the degradation of ACLY.

To explore how FBXW7 influences lipid metabolism, we referenced Khan, OM's study 25, in which FBXW7 was overexpressed in 293-T cells, then used immunoprecipitation to isolate potential gene targets, followed by mass spectrometry analysis. By comparing their results with the fatty acids metabolism gene set, ACLY was found to be present in both sets (Fig. 6A). This finding suggests that FBXW7 may regulate ACLY expression through ubiquitination, thereby impacting fatty acid synthesis and lipid droplet accumulation. To knock down FBXW7 in A-498 and 786-O cells, we transfected siRNA into the cells and conducted qRT-PCR and Western blot analyses. The results demonstrated that knocking down FBXW7 did not significantly change ACLY mRNA expression levels, a reduction in FBXW7 led to increased ACLY protein levels. Conversely, overexpression of FBXW7 using plasmids did not affect ACLY at the transcriptional level but decreased its protein expression (Fig. 6B-D). Immunofluorescence shows that FBXW7 and ACLY have a same distribution location within the A-498(Fig. 6E). Immunofluorescence also shows that it is localized in a similar position in 786-O cells (Supplementary Figures S1H). We transfected 293-T cells with FLAG-FBXW7 and MYC-ACLY plasmids, and Co-IP assays confirmed that FBXW7 can bind to ACLY (Fig. 6F-G). To determine if FBXW7 acts as a ubiquitin ligase to ACLY, promoting its degradation, we treated cells with MG-132 (40Μm, Incubating 6h), an effective proteasome inhibitor. This treatment reversed the degradation of ACLY caused by FBXW7(Fig. 6H-I). Additionally, we utilized cycloheximide (100 mg/ml) to inhibit protein synthesis in overexpressed, knocked down, and vector control cells, monitoring ACLY expression over time. We observed that ACLY's half-life shortened when FBXW7 was overexpressed, indicating decreased protein stability (Fig. 6J-K). Consistent with this conclusion, ACLY polyubiquitination increased when FBXW7 expression was elevated and decreased when FBXW7 was reduced (Fig. 6L).

### ACLY overexpression can rescue the effects of sh-LPCAT1 on ccRCC

**Figure 7:**
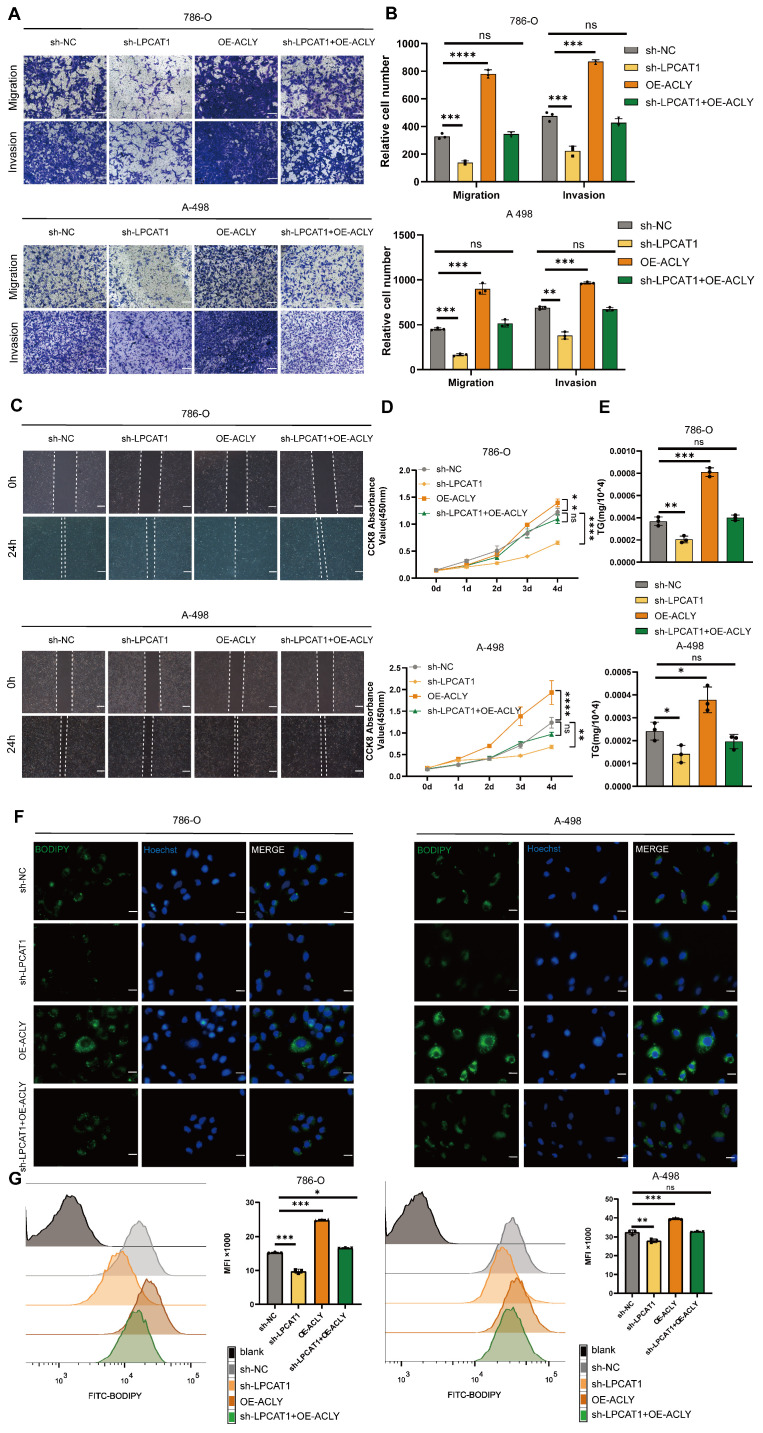
Increased expression of ACLY can counteract the effects of sh-LPCAT1 on ccRCC. **A, B**. Transwell assay was used to verify changes in the proliferation and migration abilities of transfected cells, and ImageJ was used to quantify the migrating cells (scale bar:100μm). **C**. Wound healing assay was measured to verify the cells' migration ability, and the wound healing status was observed after 24 hours. **D**. The growth curve drawn using the CCK-8 assay shows that the slowed cell proliferation caused by LPCAT1 knockdown can be alleviated by increased expression of ACLY. **E**. The triglyceride content in transfected cells was measured using a micro-TG assay kit. **F**. BODIPY 493/593 was used to detect changes in neutral lipids in transfected cells. Green indicates lipid droplets. Images were taken using an Olympus microscope(scale=20μm). **G**. Flow cytometry was used to quantitatively analyze changes in lipid droplet content in different transfected cell lines, using cells without BODIPY 493/503 as a blank control.

Our data indicate that LPCAT1 impacts FBXW7 activity via the NF-κB signaling pathway, resulting in changes in ACLY expression. ACLY is a key gene for fatty acid synthesis, and inhibiting it decreases the substrates for triglyceride synthesis, resulting in decreased lipid droplet accumulation in ccRCC. To investigate these effects, we established four cell lines: LPCAT1 knockdown (sh-LPCAT1), ACLY stably overexpression (OE-ACLY), ACLY overexpression after LPCAT1 downregulation (sh-LPCAT1+OE-ACLY), and a control group (sh-NC). To analyze the proliferation, migration, invasion, and lipid droplet levels in ccRCC cells, we used various *in vitro* assays such as CCK-8, wound healing, transwell, and BODIPY493/503. Transwell and wound healing assays revealed that ACLY overexpression greatly reduced the inhibition of migration and invasion caused by LPCAT1 knockdowns (Figure 7A-C). We found that LPCAT1 knockdown markedly reduced the growth rate of 786-O and A-498 cells, whereas ACLY overexpression substantially enhanced proliferation compared to untreated cell lines. The growth reduction due to LPCAT1 downregulation was rescued by increased ACLY expression (Fig. 7D). We utilizing a triglyceride test kit to determine the TG level of 786-O and A-498 cells, as well as in other knockdown or overexpression cell lines. Our results demonstrated that ACLY overexpression could reverse the impact of LPCAT1 knockdown on TG levels (Fig. 7E). The BODIPY493/503 assay indicated that ACLY overexpression could ameliorate the decrease in lipid droplets caused by LPCAT1 downregulation (Fig. 7F). Flow cytometry analysis of cells treated with BODIPY 493/503 exhibited the similar pattern (Figure 7G).

### LPCAT1 promotes tumor growth and lipid accumulation through ACLY

**Figure 8:**
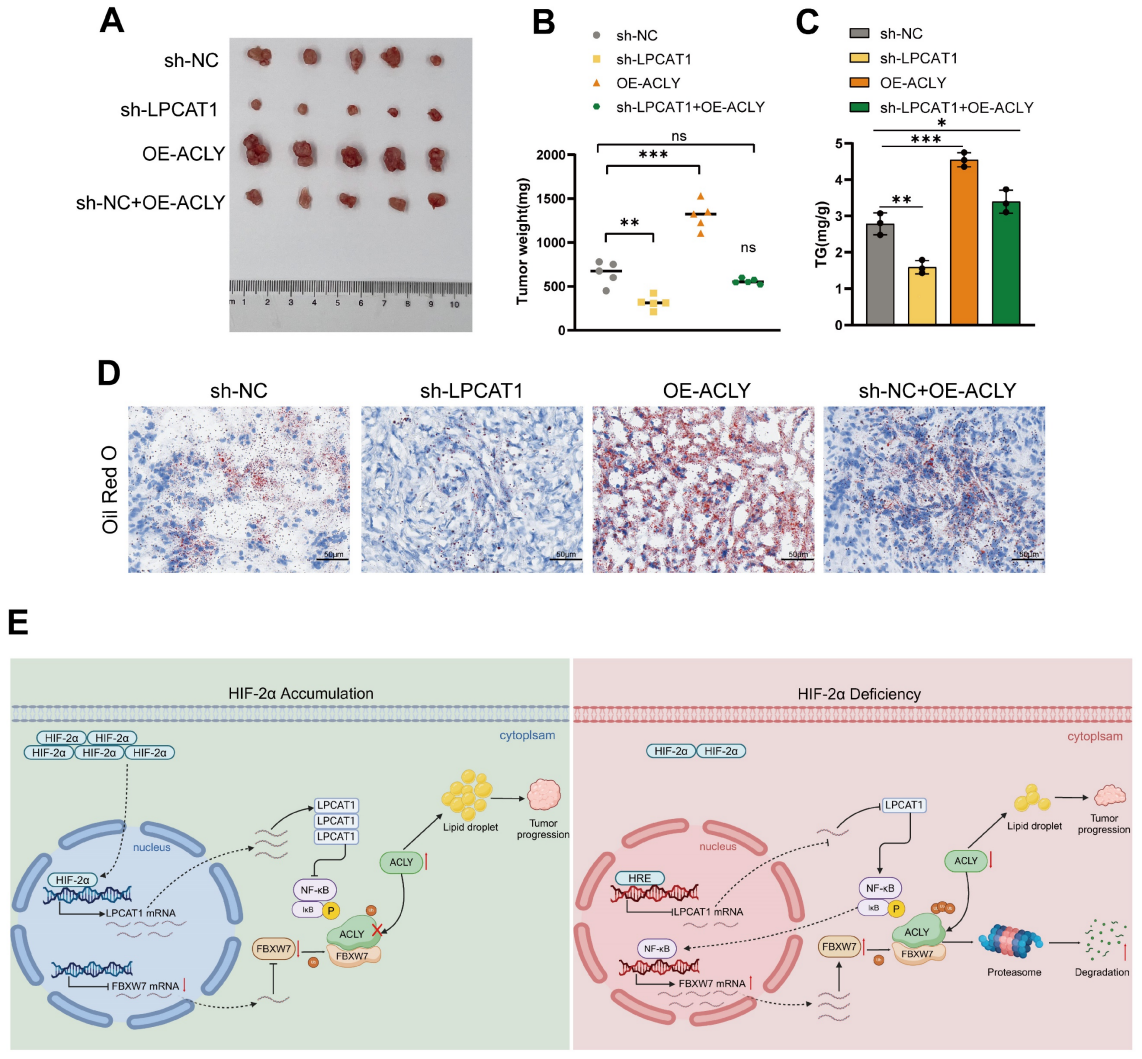
The inhibition of tumor growth and reduction in lipid accumulation caused by LPCAT1 knockdown can be reversed by the overexpression of ACLY. **A**. The transfected 786-O cells (including: sh-NC, sh-LPCAT1, and sh-LPCAT1+OE-ACLY) were transplanted subcutaneously into nude mice. Subcutaneous tumors were collected after 4 weeks (n=5). **B**. The subcutaneous tumors were weighed. **C**. The TG content in subcutaneous tumors was measured.** D**. Lipid droplets content in subcutaneous tumor samples was detected using Oil Red O staining (scale=50μm). **E**. A schematic diagram describes the proposed model of how HIF-2α-dependent LPCAT1 expression regulates ccRCC progression and lipid accumulation.

To evaluate the *in vivo* effects of LPCAT1 and ACLY, we transplanted four distinct groups of cells into the subcutaneous tissue of BALB/c-Nude mice: sh-LPCAT1, OE-ACLY, sh-LPCAT1+OE-ACLY, and sh-NC. The results revealed that LPCAT1 knockdown significantly slowed the progression of ccRCC, which was reversed by increasing ACLY expression (Fig. 8A-B). The tumor growth curve recorded the changes in tumor volume over different time periods (Supplementary Figures S1I). Both LPCAT1 and ACLY substantially affected on the triglyceride (TG) content of ccRCC *in vitro*. To verify if this phenomenon was consistent *in vivo*, we collected 100mg of tumors from each of the four groups of subcutaneous transplants and measured TG levels. Our findings demonstrated that ACLY overexpression promoted triglyceride accumulation, whereas LPCAT1 knockdown resulted in a decrease in TG content (Fig. 8C). Oil Red O staining of subcutaneously transplanted tumors in mice revealed alterations corresponding with TG levels (Fig. 8D). A schematic diagram depicts the proposed model of the main mechanisms by which HIF-2α-dependent LPCAT1 expression regulates the progression and lipid accumulation in ccRCC (Fig. 8E).

## Discussion

Current evidence demonstrates that LPCAT1 influences the proliferation, migration, invasion, and lipid reprogramming of ccRCC *in vitro* and *in vivo*. LPCAT1 also modulates the activity of FBXW7 by inhibiting the NF-κB signaling pathway. When FBXW7 is activated, the stability of ACLY, one of its binding proteins, is reduced. Since ACLY is a key gene regulating fatty acid metabolism, this ultimately leads to reduced lipid accumulation in ccRCC. Previous research has shown widespread lipid accumulation mediated by HIF-2α in renal cell carcinoma. Our study identified LPCAT1 as a direct target regulated by HIF-2α. Silencing LPCAT1 in 786-O and A-498 cell lines significantly impairs lipid synthesis, an effect that can be reversed by overexpressing ACLY, and this effect can be alleviated by increasing ACLY gene expression. LPCAT1 is overexpressed in ccRCC tissues compared to normal kidney tissues, highlighting its potential role as an oncogene in ccRCC pathogenesis.

Tumor initiation and progression are driven by metabolic reprogramming 28, which involves alterations in amino acid, glucose, and lipid metabolism 29. Lipid accumulation is a hallmark of metabolic reprogramming in ccRCC, characterized by significant lipid droplets and glycogen buildup in tumor cells 30. Studies have shown that lipid accumulation can promote tumor cell growth and invasion 31, and is also essential for maintaining energy balance, membrane biosynthesis, and lipid signaling pathways in ccRCC cells 32. Understanding the mechanisms underlying lipid metabolism reprogramming may offer new avenues for the prevention and treatment of ccRCC. Another important characteristic of renal cancer is the activation of HIF-2α. Existing research has found that HIF-2α can promote angiogenesis, tumor cell proliferation, invasion, metabolic reprogramming, and drug resistance 33. Xiao *et al.* discovered that HIF-2α promotes lipid droplet accumulation and accelerates renal cancer progression by regulating the transcription of APOL1 and maintaining endoplasmic reticulum homeostasis 34. HIF-2α directly controls the expression of the transcriptional cofactor MED15, which interacts with sterol regulatory element-binding proteins (SREBPs) to drive the expression of key lipid metabolism genes, including FASN, SCD1, and ACLY 35. Moreover, HIF-2α inhibits CPT1A transcription by binding to its promoter, thereby reducing fatty acid transport to mitochondria and promoting lipid droplet accumulation 5. Our research focuses on these two important features of lipid metabolism changes and high expression of HIF-2α in renal cancer, investigating the intersection of these processes, we aim to expand the understanding of HIF-2α-mediated lipid reprogramming and develop new strategies for the treatment of ccRCC.

LPCAT1 is primarily localized to the endoplasmic reticulum membrane, lipid droplet surfaces, and the Golgi complex membrane. It plays a critical role in regulating LysoPC synthesis, which influences endoplasmic reticulum membrane tension and lipid droplet budding 36. Additionally, LPCAT1 modulates the acylation of LysoPC 37, supplying essential phosphatidylcholine (PC) for lipid droplet fission and thereby controlling lipid droplet remodeling 38. Du *et al.* found that LPCAT1 affects the composition of phospholipids within ccRCC cells, influencing cell division and progression through the cell cycle, which in turn impacts the development and progression of ccRCC 17. Beyond its role in phospholipid metabolism, LPCAT1 also reprograms cholesterol metabolism, affecting liver cancer development 18. Interestingly, existing research has found that changes in LPCAT1 expression primarily affect the size of LDs, while changes in the content of neutral lipid droplets warrant further investigation. Our lipidomic analysis following LPCAT1 knockdown revealed significant alterations in triglyceride (TG) levels, suggesting that LPCAT1 may regulate lipid metabolism reprogramming through mechanisms other than lipid droplet volume alone.

Our study revealed that LPCAT1 knockdown activates the NF-κB signaling pathway. Previous research has shown that the expression of FBXW7 is upregulated with the activation of NF-κB 39, thereby exerting its ubiquitination function. NF-κB signaling pathway inhibition downregulates FBXW7 expression. FBXW7 has been widely studied for its significant tumor-suppressive role in various cancers 40. Several oncogenes, including c-MYC, NOTCH, and c-JUN, are ubiquitinated and degraded by FBXW7 41-43. FBXW7 regulates multiple genes associated with lipid metabolism. Li *et al.* found that FBXW7 can bind to SREBP1, promoting its degradation 44. Our data suggest that LPCAT1 may influence the proliferation, metastasis, and lipid accumulation of ccRCC in both *in vivo* and *in vitro* settings. Our study indicates that HIF-2α regulates the expression of the lipid metabolism-related gene LPCAT1. Given that ccRCC is characterized as a “metabolism-related disease,” particularly in terms of lipid metabolism, the unexplored HIF-2α/LPCAT1/ACLY pathway may present a promising therapeutic target for controlling this tumor.

## Supplementary Material

Supplementary figure.

Supplementary table.

## Figures and Tables

**Table 1:** The sample information used in this study

	Pathological pattern	Gender	Age(y)	Position	Size(cm)	TNM
						
Case 1	ccRCC, Fuhrman1-2	Female	53	Right	4.6 x 4.4 x 2.7	T1bN0M0
						
Case 2	ccRCC, Fuhrman 1	Female	42	Left	3.8 x 2.6 x 2.5	T1aN0M0
						
Case 3	ccRCC, Fuhrman 2	Male	76	Left	5.0 x 4.0 x 2.5	T1bN0M0
						
Case 4	ccRCC, Fuhrman 2	Male	58	Right	3.0 x 3.0 x 1.5	T1bN0M0
						
Case 5	ccRCC, Fuhrman 2	Male	56	Right	4.5 x 4.0x 3	T1bN0M0
						
Case 6	ccRCC, Fuhrman 2	Female	60	Right	5.6 x 5.0 x 3.0	T1bN0M0
						
Case 7	ccRCC, Fuhrman 2	Female	53	Right	6.5 x 5.0 x 3.0	T1bN0M0
						
Case 8	ccRCC, Fuhrman 2	Male	48	Right	2.8 x1. 8 x 1.2	T1aN0M0
						
Case 9	ccRCC, Fuhrman1-2	Male	59	Left	2.0x 1. 8 x 1.5	T1aN0M0
						
Case 10	ccRCC, Fuhrman 1	Male	57	Right	5.5x 4. 5x 4. 2	T1bN0M0
						
Case 11	ccRCC, Fuhrman 2	Male	54	Right	2.7 x 2.3 x 2.0	T1aN0M0
						
Case 12	ccRCC, Fuhrman 2	Male	54	Left	2.6 x 2.5 x 2.5	T1aN0M0
						
Case 13	ccRCC, Fuhrman 2	Female	56	Right	5.0 x4.0 x 3. 5	T1bN0M0
						
Case 14	ccRCC, Fuhrman 2	Male	72	Right	5.0 x 4.0 x 2.8	T1bN0M0
						
Case 15	ccRCC, Fuhrman 2	Male	71	Right	3.5 x 3.3 x 2.0	T1aN0M0
						
Case 16	ccRCC, Fuhrman 2	Male	66	Left	4.2 x 3.2 x 2.1	T1b N0M0
						
Case 17	RCC, Fuhrman 3	Female	31	Right	7.0 x 5.0 x 4.0	T1bN1M0
						
Case 18	ccRCC, Fuhrman 3	Male	63	Left	8.0 x 7.0 x 5.0	T2aN1M1
						
Case 19	ccRCC, Fuhrman1-2	Male	51	Right	7.0 x 7.0 x 6.5	T1bN0M0
						
Case 20	ccRCC, Fuhrman 2	Female	72	Left	6.0 x 6.0 x 5.0	T1bN0M0
						
Case 21	ccRCC, Fuhrman 2	Female	64	Left	5.2 x 3.6 x 3.1	T1bN0M0
						
Case 22	ccRCC, Fuhrman2-3	Male	49	Right	14.0x 9.0x 8.0	T2bN0M0
						
Case 23	ccRCC, Fuhrman 1	Female	56	Left	3.8 x3.0 x 2.5	T1aN0M0
						
Case 24	ccRCC, Fuhrman 3	Female	58	Left	7.5 x 6.7 x 5.8	T3bN0M0
						
Case 25	ccRCC, Fuhrman 3	Female	77	Right	6.0 x 5.0 x 2.0	T3bN0M0

## Abbreviations

ccRCC: clear cell renal cell carcinoma
RCC: renal cell carcinoma
VHL: von Hippel-Lindau
HIFs: hypoxia-inducible factors
LPCAT1: Lysophosphatidylcholine Acyltransferase 1
FBXW7: F-Box/WD Repeat-Containing Protein 7
ACLY: ATP Citrate Lyase
GEO: Gene Expression Omnibus
TCGA: The Cancer Genome Atlas Program
CPTAC: The Clinical Proteomic Tumor Analysis Consortium
