# Supplementary material for: HIF-2α/LPCAT1 orchestrates the reprogramming of lipid metabolism in ccRCC by modulating the FBXW7-mediated ubiquitination of ACLY
Source: Int J Biol Sci. 2025 Jan 1;21(2):614–31. doi: 10.7150/ijbs.103032 (PMC11705634; doi:10.7150/ijbs.103032)
Supplement: Supplementary file 1 — Supplementary figure. [file ijbsv21p0614s1.pdf]

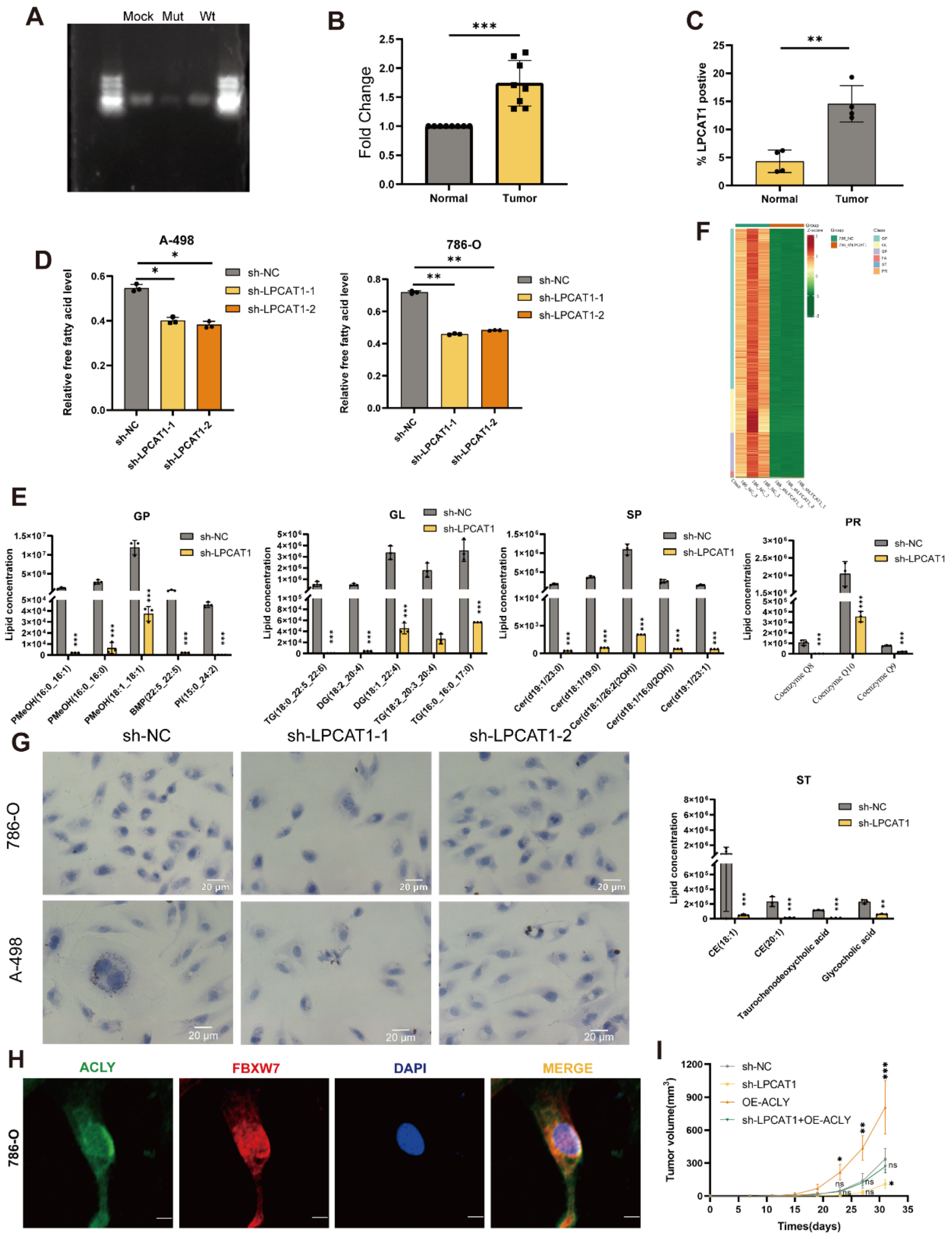

Supplementary Figure:

A. Gel electrophoresis shows the levels of LPCAT1 after ChIP-qPCR amplification in Mock, Mutant, and Wild-type samples. **B**. Quantitative analysis of LPCAT1 protein levels in tumor and normal tissues using Western blot assay. **C**. Quantitative analysis of LPCAT1 positive rates in tumor and normal tissues as determined by IHC. **D.** Detection of fatty acid content in 786-O and A-498 Cells after LPCAT1 Knockdown **E**. Top five lipid components showing the greatest changes in abundance according to LC/MS results. **F**. Heatmap illustrating alterations in lipid composition in 786-O cells after sh-LPCAT1 treatment. **G**. Oil Red O staining reveals

changes in lipid droplet content following sh-LPCAT1 treatment. **H**. Immunofluorescence displays the localization of ACLY and FBXW7 in cells following transfection with MYC-ACLY and FLAG-FBXW7 plasmids, using FLAG and MYC antibodies for incubation. **I.** The tumor growth curve recorded the changes in tumor volume over different time periods.
